# Microcavitation: the key to modeling blast traumatic brain injury?

**DOI:** 10.2217/cnc-2017-0011

**Published:** 2017-08-01

**Authors:** Christian Franck

**Affiliations:** 1 School of Engineering, Brown University, Providence, RI, USA

**Keywords:** cavitation, neuronal injury, primary blast injury, traumatic brain injury

Traumatic brain injuries (TBIs) are a significant source of deaths and disabilities worldwide with an associated healthcare burden in the billions of dollars [1]. Brain injuries generally result from either direct impact, blast or rapid acceleration and deceleration of the brain, and their severity is graded neurosymptomatically from mild to severe using the Glasgow Coma Scale. While these injuries, which in their mild form include concussions, are generally initiated by mechanical stress waves traveling through the brain resulting in exceeding tissue damage quantified as either compressive, tensile or shearing strains [2–4], blast TBIs have a slightly different origin, and as thus their injury mechanism and pathology remain an active topic of research [5,6].

In blast waves generated from explosions, including improvised explosive devices (IEDs) [6,7], the initial blast-generated shock wave profile features a sudden increase in pressure, often referred to as overpressure, followed by a low magnitude long-range negative pressure tail [5,7]. This profile is significantly different from most civilian blunt head impact scenarios, which, at least initially, are almost entirely composed of fast traveling pressure waves [5]. These shock-like pressure profiles introduce significant pressure changes across the brain on the order of a micro- to submilliseconds, whereas typical blunt trauma stresses change over the course of milliseconds and above.

The classification of blast TBI has its own categorization from primary to quaternary blast injury [6]. Secondary to quaternary blast injuries have correlates in the civilian world whereas primary blast injuries that are classified by the interaction of the blast wave itself with the brain are unique to military and law enforcement personnel. Details of the origin of the injury and its pathology have remained elusive. Part of the challenge in dissecting the details of blast injury lies in the complex physical interaction between the fast moving pressure wave and the compliant brain. Furthermore, our understanding of the deformation behavior of soft brain tissue and its relationship to specific neuropathologies is still in its infancy.

Although the initial blast wave is generally a pure pressure wave, it can turn into part pressure and part shear wave upon encountering the complex geometry of the human head and brain. While the traversing pressure wave will cause the tissue to undergo changes in volume, the shear wave can generate significant changes in shape, or shearing strains. In addition, part of the original pressure wave can reflect off a boundary of lower impedance, which is marked by either changes in tissue density or compliance, resulting in a negative, tensile, pressure reflection wave [8]. While a significant body of work has begun to detail the interaction of the compressive part of the wave with brain tissue and its cells [9–12], we will focus our attention here on the negative, or tensile character of the pressure wave. Recent experimental and finite element investigations simulating blasts to the head and brain have shown that these negative, tensile region of pressure can give rise to the phenomenon known as cavitation, which generally denotes the formation of vapor bubbles from a liquid [13,14]. Cavitation damage is a well-appreciated phenomenon, first studied in mechanical pumps and underwater propulsion systems, and also found in the prey stunning capability of the snapping pistol shrimp [15–17]. In addition, many human medical applications including kidney stone removal via lithotripsy and tissue ablation via histotripsy make use of the power of cavitation [18,19]. Yet when it comes to the brain, cavitation remains to be fully characterized.

Because of the highly localized and short-lived nature of cavitation, documentation of the phenomenon itself has been challenging and efforts are still underway to prove its existence in the human brain during blast exposures. However, increasing mounting evidence, in particular in the last few years, has provided us with a better understanding that inertial microcavitation in the brain might indeed be a real possibility [13,20–23]. For example, Goeller *et al.* demonstrated the generation of cavitation bubbles at the back, or countercoup, region of an elastomeric surrogate brain during a frontal blast exposure [13]. More recently, Salzar *et al.* provided intracranial pressure readings resembling cavitation events inside of postmortem human subject heads. Although no visual confirmation was presented in their work, inference from the pressure measurements and finite element calculations lend credence to the possibility of cavitation occurring during blast exposure [22].

In a detailed report, Baughman Shively *et al.* provided the first postmortem human brain tissue analysis specifically focused on the effects of primary blast exposure, which paints a dramatically different injury pathology than blunt trauma (concussive) injury [21]. Moreover, their analysis shows significant cell and tissue damage highly localized to internal brain interfaces capable of producing reflection and tensile waves. Close inspection of the extent of the localized tissue damage shows length scales on the order of several hundred micrometers, which is on a similar order of damage produced by submillimeter- or micrometer-sized cavitation bubbles, or microcavitation bubbles.

These results, along with several other experiments on human brain surrogates of ranging realism demonstrating the existence of cavitation at pressures around -1 atmospheres (or roughly -100 kPa), are pointing to suggestive evidence that microcavitation might indeed be a possible injury mechanism of not just blast but also possibly blunt head trauma [13,20,22,23]. Works by several groups have shown that pressures near the estimated cavitation threshold of -1 atmosphere can be generated in head impacts sustained from sports-related or vehicular incidents, even in the absence of an explosive pressure wave [24,25]. If this was the case, microcavitation could play an even bigger role in shaping our understanding of the origin of brain trauma, not just in blast but also in concussive injuries. This begs the question of what the pathology of microcavitation-induced brain injury might look like.

To address this question, we turn to the laboratory where several researchers have begun to map out the injury pathology, stress and strain maps of cavitation-inflicted tissue and cell damage [20,23]. By incorporating advanced mechanical engineering models of cavitation alongside carefully executed *in vitro* experiments in brain surrogate materials, a glimpse of just how devastating cavitation can be on cells in the brain can be gleamed. For example, as a cavitation bubble grows, it displaces the material around it, thereby exerting significant compressive strains on cells in its immediate vicinity. Following the growth comes the collapse of the bubble, which is marked by the emanation of local shock waves, temperatures in excess of 6000 K with accompanying pressures over 1000 atmospheres [26], and in many cases high-energy jets that are known to destruct even the strongest man-made materials [5,15]. Yet, these impressive growth and collapse characteristics typically only last a few to tens of nanoseconds, and remain highly localized, which has made their detection on traditional biopsies and medical imaging devices difficult. Besides the large material strains, which can reach estimated magnitudes of up to 500% in compression and up to 300% in tension, are the extremely high loading, or strain rates. Since many inertial cavitation events feature several collapse and expansion phases over the course of less than a millisecond, the adjacent tissue experiences loading rates as fast as impact strikes of asteroids, far beyond the blunt trauma and ballistic regimes. It might thus not be surprising that cavitation-induced injury might present itself as a new pathology paradigm for cells in the brain. Efforts by various groups are currently underway for providing high-resolution pathology maps of this new type of cellular injury. In addition to understanding the cavitation damage and resulting neuropathology to the brain tissue itself, several studies have been investigating disruptions in the blood–brain barrier due to cavitation and shock-induced imploding nanobubbles [27,28]. In a recent perspective, for example, Adhikari *et al.* provide a detailed summary on the potential damage mechanism of shock wave-induced nanobubble implosions incorporating cellular lipid bilayers, which are integral to the vascular endothelium in the brain [29].

The eventual road to success will require the integration of knowledge gained from a variety of experimental and numerical approaches spanning the *in vitro* to *in vivo*, and neuroscience, biological and engineering worlds. While animal and cadaver experiments will provide the highest degree of physiological relevance, *in vitro* and finite element-based modeling approaches will provide the best solution for detailing the initial injury pathologies and locations within the brain. In particular, when it comes to the cellular injury response in neurons and other brain cells, data show just how sensitive cells are to local tissue strains and their loading rates [2,4]. These measurements when extracted from *in vitro* models and combined with numerical models can provide predictive estimates for *in vivo* screening, which should provide the *in vivo* world with the necessary parameters for successful diagnosis and detection. In short, this synergistic and highly integrated approach spanning multiple disciplines is poised to accelerate our understanding of the pathology and origin of blast injury and define the role of microcavitation in a perhaps much larger context of traumatic brain injuries. Thus, it is conceivable, at least hypothetically, that inertial microcavitation may not only be the defining injury signature of the recent conflicts in Iraq and Afghanistan, but play a much larger role in defining the nature of brain injury including sports-related and vehicular injuries. And finally, from an engineering perspective, these advances in injury characterization will provide the necessary ingredients for the development of new and advanced head impact protection standards and solutions.
